# ESBL/AmpC-Producing *Escherichia coli* in Wild Boar: Epidemiology and Risk Factors

**DOI:** 10.3390/ani11071855

**Published:** 2021-06-22

**Authors:** Nicoletta Formenti, Stefania Calò, Giovanni Parisio, Flavia Guarneri, Laura Birbes, Alessandra Pitozzi, Federico Scali, Matteo Tonni, Federica Guadagno, Stefano Giovannini, Cristian Salogni, Adriana Ianieri, Silvia Bellini, Paolo Pasquali, Giovanni Loris Alborali

**Affiliations:** 1 Istituto Zooprofilattico Sperimentale della Lombardia e dell’Emilia Romagna “Bruno Ubertini”, Via Bianchi 7/9, 25124 Brescia, Italy; stefania.calo@izsler.it (S.C.); giovanni.parisio@izsler.it (G.P.); flavia.guarneri@gmail.com (F.G.); laura.birbes@izsler.it (L.B.); alessandra.pitozzi@izsler.it (A.P.); federico.scali@izsler.it (F.S.); matteo.tonni@izsler.it (M.T.); federica.guadagno@izsler.it (F.G.); stefano.giovannini@izsler.it (S.G.); cristian.salogni@izsler.it (C.S.); silvia.bellini@izsler.it (S.B.); giovanni.alborali@izsler.it (G.L.A.); 2 Dipartimento di Scienze degli Alimenti e del Farmaco, Università di Parma, Parco Area delle Scienze 27/A, 43124 Parma, Italy; adriana.ianieri@unipr.it; 3 Dipartimento di Sicurezza Alimentare, Nutrizione e Sanità Pubblica Veterinaria, Istituto Superiore di Sanità, Viale Regina Elena 299, 00161 Roma, Italy; paolo.pasquali@iss.it

**Keywords:** *Sus scrofa*, *bla*_CTX-M_, human population density, *bla*_TEM_, age class, environmental contamination

## Abstract

**Simple Summary:**

Antimicrobial resistance (AMR) represents a complex global issue due to the many factors involved. Extended-spectrum β-lactamase and AmpC (ESBL/AmpC)-producing *Escherichia coli* deserves attention for its broad repercussions on public health. Moreover, wild host species are of interest, particularly wild boar. Indeed, the constantly increasing population densities and the limited data on AMR in this species lead to health risks where spatial overlap with humans and domestic animals occurs. Therefore, 1504 wild boar fecal samples were analyzed to investigate ESBL/AmpC-producing *E. coli* and the effects of host-related factors and of human population density on their spread. A high prevalence of ESBL/AmpC-producing *E. coli* emerged in wild boar, species not treated with antibiotics, supporting that infection may be acquired through environmental contamination, whether of human or animal origin. Young animals were more colonized than older ones, demonstrating higher susceptibility as seen in domestic animals. Moreover, a positive association recorded between frequency of the TEM resistance gene and human population density suggests that spatial overlap may influence the infection in wild boar. Further analyses would be desirable to investigate the origin of the recorded environmental contamination, although a role of wild boar as a maintenance host of AMR strains emerged.

**Abstract:**

The complex health problem of antimicrobial resistance (AMR) involves many host species, numerous bacteria and several routes of transmission. Extended-spectrum β-lactamase and AmpC (ESBL/AmpC)-producing *Escherichia coli* are among the most important strains. Moreover, wildlife hosts are of interest as they are likely antibiotics free and are assumed as environmental indicators of AMR contamination. Particularly, wild boar (*Sus scrofa*) deserves attention because of its increased population densities, with consequent health risks at the wildlife–domestic–human interface, and the limited data available on AMR. Here, 1504 wild boar fecal samples were microbiologically and molecularly analyzed to investigate ESBL/AmpC-producing *E. coli* and, through generalized linear models, the effects of host-related factors and of human population density on their spread. A prevalence of 15.96% of ESBL/AmpC-producing *E. coli*, supported by *bla*_CTX-M_ (12.3%), *bla*_TEM_ (6.98%), *bla*_CMY_ (0.86%) and *bla*_SHV_ (0.47%) gene detection, emerged. Young animals were more colonized by ESBL/AmpC strains than older subjects, as observed in domestic animals. Increased human population density leads to increased *bla*_TEM_ prevalence in wild boar, suggesting that spatial overlap may favor this transmission. Our results show a high level of AMR contamination in the study area that should be further investigated. However, a role of wild boar as a maintenance host of AMR strains emerged.

## 1. Introduction

Antimicrobial resistance (AMR) represents a global health problem that involves humans, animal species and ecosystems [1,2,3]. Animals can play a role as reservoir of pathogenic and non-pathogenic antimicrobial-resistant microorganisms that, through direct contact or toward the food chain, can contribute to the spread and/or maintenance of AMR [4].

*Escherichia coli* is one of the most important bacteria that contributes to the complexity of AMR, and among its strains, those producing β-lactamases, extended-spectrum β-lactamases (ESBLs) and other β-lactamases such as AmpCs encoded by plasmid-located genes are of particular interest. Indeed, resistance to most beta-lactam antibiotics, including third and fourth-generation cephalosporins, can limit treatment options in case of infection [5] and lead to major public health concerns [6]. Authors reported 8750 deaths ascribed to *E. coli* resistant to third-generation cephalosporins in the European Union and European Economic Area during 2015, and comparing these data with others from 2007, an approximately 4-fold increase was detected [7]. The presence of *E. coli* ESBL and AmpC has been reported in several animal species [7,8,9], but, among them, wildlife is of particular epidemiological interest [10].

Wild species are generally not treated with antimicrobials and may acquire AMR bacteria or antimicrobial residues just through food and water in environments contaminated by domestic animals or humans [11]. Indeed, wildlife in anthropized environments or in close contact to humans or agricultural areas showed higher levels of AMR [12]. Thus, wildlife can be assumed as an important indicator for assessing the environmental spread of antimicrobial bacteria or AMR genes [4].

Among free-range species, wild boar (*Sus scrofa*) has been of high interest because of its role of an ‘invasive species’, the very close phylogenetical similarity to pigs (*Sus scrofa domesticus*) and the great increase of its population densities [13]. The continuous expansion of the habitat ranges of this species in close proximity to farms and pastures (anthropized environment) leads to opportunities for spatial overlap and contact with other species favoring health risks of sharing infections across the wildlife–domestic animal–human interface [14], such as antimicrobial resistant *E. coli*. Indeed, the use of antibiotics in both veterinary and human medicine and the known relationship between usage of antimicrobials and the occurrence of resistance in bacterial isolates in manure or manure contaminated surface water leads to AMR risks for wild boars in anthropized environments and highlights the role of drug resistance “conductor” of the environment [11,15]. *E. coli* is constantly present both in the digestive tract and in the environment, e.g., in water and soil [15], and this can contribute to the transfer of resistant genes between strains and to the increase in the drug resistance in environmental bacteria [15], an issue that is not yet sufficiently researched. Moreover, wild boar can act as a potential, but still to be defined, reservoir of AMR [16], maintaining strains/AMR genes through direct or indirect contact within its populations. This issue of the likely transmission of these pathogenic strains to humans through handling or consumption of contaminated game meat [7,17] should be taken into account. Nevertheless, data on ESBL and AmpC-producing *E. coli* in wild boar are still limited, especially with respect to factors that may influence its spread or the potential health risks related to the contact of these populations with humans or domestic animals.

Therefore, we carried out an epidemiological investigation of ESBL/AmpC-producing *E. coli* in free-ranging wild boar from Northwest Italy and aimed to evaluate (i) the prevalence within the population and (ii) the effects of host-related factors and of human population density on the spread of these pathogenic bacteria.

## 2. Materials and Methods

### 2.1. Study Area

**Figure 1:**
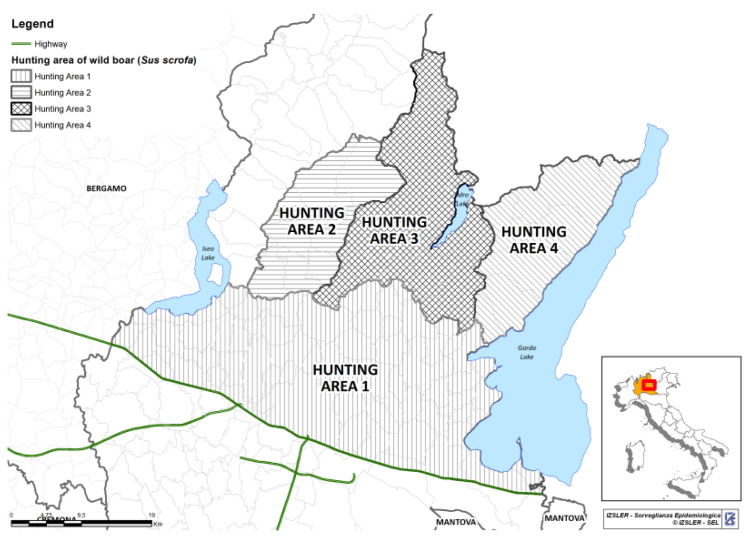
Map of the four hunting areas that formed the study area.

The study area was in Northern Italy within the easternmost part of the Lombardy region (Province of Brescia; 45°32′20″ N, 10°13′10″ E). The territory includes four wild boar hunting areas (Figure 1). From the official data on hunting activities provided by the local hunting office, consistent hunting behavior (dependent on the number of hunters and hunting days) was assumed among hunting areas and years. For this reasons, Chiari et al. [18] used the total number of wild boar hunted per year as an approximation of wild boar abundance and calculated a relative abundance index to take into account the different sizes of hunting areas, scaling abundance to district areas in km^2^ (Table 1). For each hunting area, the human population density was calculated based on data from the Italian Statistical Institute (ISTAT). Briefly, the number of people inhabiting the municipalities in the hunting areas was collected. When only a part of a municipality was included in a hunting area, the population was divided based on the percentage of the area in the municipality assuming that people were evenly distributed. To calculate the human population density within each hunting area, the total population present was scaled to district areas in km^2^ (Table 1).

### 2.2. Sampling

During three hunting seasons (2017/2018, 2018/2019 and 2019/2020; a total of 1504), wild boar fecal samples were collected through the regional health monitoring program on free-ranging animals in the Lombardy region (D.d.g. 5 December 2012—no. 11358). By hunting season, 34.91% of wild boars were hunted in 2017–2018, 25.33% in 2018–2019 and 39.76% in 2019–2020. For each wild boar, gender, age and the hunting area (HA) were registered. Of the hunted wild boars, 53.32% were females and 46.68% males. The age of wild boar was evaluated based on tooth eruption patterns [18], and individuals were divided into “young” (≤12 months), 22.94% of the sample; “sub-adult” (13–24 months), 24.47% of the sample and “adult” (>24 months), 52.59% of the sample. Based on hunting areas, 56.91% of wild boars were hunted in HA 4, 33.64% in HA 1, 5.25% in HA 3 and 4.19% in HA 2.

### 2.3. Isolation and Identification of ESBL/AmpC E. coli

The identification of β-lactamase-producing *E. coli* was performed through a double synergy diagnostic method. Specifically, 1 g of feces was diluted in 9 mL (1:10 dilution) of brain–heart infusion (BHI) broth supplemented with 1 mg/L cefotaxime for a pre-enrichment phase. After an overnight incubation, a drop of the BHI broth was used to inoculate MacConkey agar supplemented with 1 mg/L cefotaxime [19]. Positive growths were identified as pink to dark-pink colonies (lactose +), tested for activity of indole (+) and citrate (−), and one of these was selected for further molecular characterization. On the basis of staining and morphological characteristics, suspect colonies were selected and subjected to subsequent molecular characterization. A single bacterial colony from each phenotype-positive sample was resuspended in 250 μL of DNase-RNase free water, and DNA was extracted by lysis boiling (98 °C for 10 min). Identification of *E. coli* was conducted by PCR phylogenetic group analysis according to Clermont et al. [20]. This is a quadruplex method that can detect the eight *E. coli* phylogroups A, B1, B2, C, D, E, F and clade I and can exclude other strains with typical *Escherichia* phenotype, i.e., *E. albertii* and *E. fergusonii*.

### 2.4. Analysis of Resistance Genes

The detection of resistance genes was performed with a panel of reactions. Specifically, multiple PCR was used for the identification of *bla*_CTX-M_ [21]; single PCRs were used for *bla*_SHV_ [22], *bla*_TEM_ [23] and *bla*_CMY_ [24]. All the PCR gene targets, thermal profiles and primer sequences used for the detection of ESBL/AmpC *E. coli* are described in Table S1. In Figure S1 the agarose gel electrophoresis analysis of each specific genes, *bla*_CTX-M_, *bla*_SHV_, *bla*_TEM_ and *bla*_CMY_, is shown. All the amplicons found to be positive for *bla*_TEM_ and *bla*_SHV_ were sequenced [25].

### 2.5. Statistical Analysis

The analysis can be split into three components: descriptive analysis of hunted wild boar, estimation of the apparent prevalence (p) with the corresponding confidence intervals estimated using an exact method and analysis of factors associated with prevalence. We used logistic regression models (GLM) [26] to define the effects of host-related explanatory variables (gender, age class, hunting season, hunting area, wild boar abundance) and environment-related explanatory variables (human population density) on the probability of wild boar to be positive for ESBL-/AmpC-producing *E. coli*. To investigate the effects of the same explanatory variables on AMR genes spread, we modeled each gene of microbiological positive subjects using a Binomial GLM. The models were compared using the likelihood ratio test (LRT). A post-hoc Tukey test was used to perform pairwise comparisons when statistically significant factors were detected in the models. Statistical analyses were carried out using R software version 4.0.3 [27], specifically the binom and emmeans packages. Results were considered statistically significant if the *p*-value (*p*) was less than 0.05.

## 3. Results

The phylogenetic analysis showed the presence of 240 *E. coli* distributed among all the eight phylo-groups.

Specifically, the distribution of the studied strains in the different phylogenetic groups was as follows: B1 (*n* = 63, 26.25%), A (*n* = 59, 24.58%), F (*n* = 31, 12.92%), C (*n* = 28, 11.67%), B2 (*n* = 21, 8.75%), E (*n* = 18, 7.50%), D (*n* = 16, 6.67%), clade I (*n* = 4, 1.67%). The overall microbiological prevalence of ESBL/AmpC-producing *E. coli* in wild boars was 15.96% (240/1504, 95% C.I. 14.14–17.91%) (Table 2). Statistical analysis showed effects of age class, hunting season and hunting area on the probability of testing positive. In particular, young animals were more colonized by *E. coli* ESBL/AmpC than adults (*p* = 0.0207) and sub-adults (*p* = 0.0098). In wild boars hunted during 2017–2018, the probability of being positive was significantly higher compared to in 2018–2019 (*p* = 0.0232) and 2019–2020 (*p* = 0.0015). Wild boars hunted in HA 3 were more colonized by *E. coli* ESBL/AmpC than those of HA 4 (*p* = 0.0033) (Table 2). No other significant differences were found (*p* > 0.05).

According to AMR resistance genes, the overall prevalence of *bla*_CTX-M_ in *E. coli* isolates was 12.3% (185/1504, 95% C.I. 10.68–14.07%). Statistical models showed the effects of hunting season and age class on the presence of this gene (Table 3). Indeed, in strains isolated from young animals, the probability of detecting *bla*_CTX-M_ was higher than in *E. coli* of sub-adults (*p* = 0.0197) and adults (*p* = 0.0245) (Table 3). Wild boars hunted during 2017–2018 had a significantly higher prevalence of this gene than animals of 2019–2020 (*p* = 0.0005). No other statistically significant differences emerged (*p* > 0.05).

Concerning *bla*_CMY_ resistance gene, the overall prevalence in *E. coli* isolates was of 0.86% (13/1504, 95% C.I. 0.46–1.47%). Statistical analysis did not reveal any significant effect (Table S2). Regarding to *bla*_SHV_ resistance gene, the apparent prevalence recorded in *E. coli* isolates was of 0.47% (7/1504, 95% CI 0.19–0.96%). Sequence analysis of the amplicons revealed the presence of SHV-12 (7/7). Sequences were deposited in NCBI GenBank with accession numbers from MZ026052 to MZ026058. No significant effects emerged from the statistical models (Table S3).

Concerning *bla*_TEM_, the apparent prevalence was of 6.98% (105/1504, 95% C.I. 5.74–8.39%). Analysis of the amplicon sequences (*n* = 98, seven cases were non-typeable) revealed the presence of TEM-1 and its variants (91/98), TEM-33 (1/98), TEM-135 (2/98) and TEM-176 (3/98). Additionally, a novel *bla*_TEM_ allele, named TEM-244 was detected in one isolate (1/98). Sequences were deposited in NCBI GenBank with accession numbers from MZ026059 to MZ026156. Hunting season and human population density influence the probability of detecting the gene: animals hunted during 2017–2018 had a significantly higher prevalence of *bla*_TEM_ than those of 2018–2019 (*p* = 0.0186) and 2019–2020 (*p* = 0.0368) (Table 4). A statistically significant positive association was observed between *bla*_TEM_ and human population density: as the human population density increases, the probability of wild boar to being positive for *bla*_TEM_ increases by 23% (*p* = 0.0143). No other statistically significant differences emerged (*p* > 0.05).

## 4. Discussion

The present study revealed a high prevalence of ESBL/AmpC-producing *E. coli* in free-range wild boar. Young animals were more colonized by ESBL/AmpC strains, and particularly by the *bla*_CTX-M_ resistance gene, than older subjects. Wild boar hunted during hunting season 2017–2018 showed a higher prevalence of ESBL/AmpC strains, *bla*_CTX-M_ and *bla*_TEM_ than animals of 2018–2019 and 2019–2020. The increasing of human population density leads to an increase of prevalence of *bla*_TEM_ in wild boar.

In this study the most frequent ESBL/AmpC-producing *E. coli* phylogenetic groups were B1 (26.25%, 63/240) and A (24.58%, 59/240), in accordance with Holtmann et al. [7] (B1 = 54.55%, 12/22; A = 4/22, 18.18%). The results of both ESBL/AmpC-producing *E. coli* and AMR genes recorded are consistent with previous reports for this species [28,29,30]. Although our prevalence of ESBL/AmpC-producing *E. coli* (15.96%, 240/1504) appears higher than those previously recorded in wild boars by Wasyl et al. [1] (2.7%, 9/332), Literak et al. [31] (2%, 5/293), Bonardi et al. [32] (0.9%, 1/108) and Holtmann et al. [7] (5.9%, 22/375), the lack of a standardized diagnostic method makes difficult the comparison of results of different studies [12]. However, *bla*_CTX-M_ (12.3%, 185/1504) was confirmed as the most frequent among AMR genes as reported by Literak et al. [31] (80%, 4/5) and Holtmann et al. [7] (68.18%, 15/22). The findings of TEM-1 (92/98) and SHV-12 (7/7) as the most frequent variants of *bla*_TEM_ and *bla*_SHV_ are consistent with the results of Holtmann et al. [7] (TEM-1 = 5/7; SHV-12 = 4/4). Considering that free-range species should be free of antibiotics/AMR, the recorded prevalence suggests that the acquisition of ESBL/AmpC-producing *E. coli* or of AMR genes occurs through direct contact with the environment, soils or surface water (feeding and drinking) contaminated with livestock/pig manure [7,33]. A possible explanation for the differences in prevalence recorded between our study and the previous ones could be related to a higher AMR environmental contamination of our study area. Indeed, all samples were collected in an area with a high density of farms, and Italy is one of the largest consumers of veterinary antimicrobials in Europe [34], even in age groups such as finisher pigs, where the use of antimicrobials should be low [35]. In this regard, the intrinsic characteristics of the wild boar’s diet, an opportunistic omnivore that can change its diet drastically depending on the availability of food, should also be taken into account. Although currently no data on the prevalence of AMR in domestic animals living in spatial overlap with wild boar are available to be included in the analysis, further investigations will address this aspect.

Statistical models showed a higher prevalence of *E. coli* ESBL/AmpC (20.58%) and *bla*_CTX-M_ (16.52%) in young animals compared to sub-adults (ESBL/AmpC = 13.32%, *bla*_CTX-M_ = 10.05%) and adults (ESBL/AmpC = 15.17%, *bla*_CTX-M_ = 11.5%). To the best of our knowledge, in the only available reference in which an age comparison of prevalence of these strains was performed for wild boar, no significant differences were registered [7]. However, the fact that in pig farms piglets have an increased susceptibility to *E coli* strains, which can cause severe enteritis especially when they are only a few weeks old [36], suggests that young wild boar may also have an increased susceptibility to this infection. Furthermore, similarly to domestic animals, young individuals may have a greater susceptibility to these strains due to a not yet fully developed intestinal microflora and may be more susceptible to new bacterial colonization [37]. In order to better understand the transmission of *E. coli* that occurred in young wild boars, an in-depth analyses of virulence factors, i.e., adhesins (K88, K99, F41, 987P and F18), of isolated *E. coli* would be useful to determine whether these strains were part of the normal microflora or could potentially cause diseases in domestic pigs.

The difference in prevalence of ESBL/AmpC strains (2017/2018 = 20.57%, 2018/2019 = 14.44%, 2019/2020 = 12.88%), *bla*_CTX-M_ (2017/2018 = 16.00%, 2018/2019 = 12.60%, 2019/2020 = 8.86%) and *bla*_TEM_ (2017/2018 = 9.33%, 2018/2019 = 5.51%, 2019/2020 = 5.85%) recorded between hunting seasons could be associated with both the role of the environmental indicator of wild boar and that of the “conductor” in the transmission of pathogenic strains/resistance genes of the environment [2]. The suggested hypothesis is that until 2017, the natural ecosystem of wild boar was more contaminated by resistant bacteria than in the subsequent years. However, with our data we cannot discriminate which mechanism actually occurs and what origin this contamination had, whether human or animal, in light of the surprising rapidity with which this change in trend of prevalence between hunting seasons occurred. However, the occurrence of *bla*_TEM_ in *E. coli* of wild boar being positively related to human population density suggests that this spatial overlap, particularly due to feeding with anthropogenic food sources and waste, can expose wild boar to traces of antimicrobial substances or directly to resistant bacteria of human origin [7]. In the late 1990s, when most ESBLs were mutants of the classical TEM-1, -2 and SHV-1 enzymes [38], *bla*_TEM_ was the most frequent isolated among nosocomial strains [39], being also detected in commensal strains from healthy adults [40]. From the 2000s the *bla*_CTX-M_ genes became the dominant enzymes in human populations [41]. Indeed, Livermore et al. [38] reported that in Italy, ESBL prevalence increased steadily from 0.2% in 1999 to 1.6% in 2003, with this change largely attributable to CTX-M-β-lactamase-positive isolates, which comprised 38% of all ESBL producers in 2003 compared with 12% in 1999. Our results may appear surprising considering the findings of Holtmann et al. [7] who showed a positive relationship between ESBL strains and human population density. Nevertheless, as *bla*_TEM_ still occurs in the most clinically relevant enterobacterial species [39] with the emergence of its new variants [42], and given that no data are currently available on the prevalence of *bla*_CTX-M_ in the human population of our study area, the hypothesis that *bla*_CTX-M_ may not yet be as widespread in the human population and therefore does not have a direct effect on wild boar could be suggested.

The high prevalence of ESBL/AmpC-producing *E. coli* recorded in wild boar in this study leads to strategies for reducing and preventing the spread of resistant bacteria in the environment and between species. As the main concern of AMR is related to the over-use of antimicrobials [43], the reduction of antibiotic administration in domestic animals should be the starting point. In addition, farm biosecurity standards and infrastructure should be improved in order to minimize the impact of animal husbandry manure in the environment and to prevent the related potential spreading of AMR bacteria. Attention should also be paid to preventing wild boar feeding with anthropogenic waste or food sources that can further expose them to traces of antimicrobials or directly to resistant bacteria of human origin.

## 5. Conclusions

The high prevalence of ESBL/AmpC-producing *E. coli* recorded in this study shows the potential role of wild boar as a maintenance host of AMR strains. However, the creation of historical data series would be desirable in order to monitor and better evaluate these temporal trends in wild boar. In addition, the role of this species as indicator of environmental contamination, supported by the recorded positive association between *bla*_TEM_ and human population density, leads to an in-depth analysis that also includes domestic animals. Indeed, through a “One Health” approach, further specific sampling should be carried out at both farm and human/environmental levels within hunting areas to assess prevalence of these strains/AMR genes and to evaluate whether their presence actually influences their spread in wild boar.

## Figures and Tables

**Table 1:** Wild boar (*Sus scrofa*) abundance, number of people and human population density in the study area.

Hunting Areas (HA)	Area (km^2^)	Mean Number of Hunted Wild Boar (*Sus scrofa*)	Abundance (Wild Boar/km^2^)	No. of People	Human Density
HA 1	887.45	272	0.31	518,424	584.17
HA 2	231.62	323	1.40	25,036	108.09
HA 3	401.15	132	0.33	24,867	61.99
HA 4	272.21	403	1.48	12,659	46.5

**Table 2:** Prevalence of ESBL/AmpC-producing *E. coli* in wild boars by sex, age class, hunting season and hunting area.

Factors		Positive	Total	Prevalence %	95% C.I. *
Sex	Female	129	802	16.08	13.61–18.81
	Male	111	702	15.81	13.19–18.72
Age class	Young	71	345	20.58	16.44–25.24
	Sub-adult	49	368	13.32	10.02–17.22
	Adult	120	791	15.17	12.74–17.86
Hunting season	2017–2018	108	525	20.57	17.19–24.29
	2018–2019	55	381	14.44	11.06–18.37
	2019–2020	77	598	12.88	10.30–15.83
Hunting area	HA 1	83	506	16.40	13.28–19.92
	HA 2	11	63	17.46	9.05–29.10
	HA 3	22	79	27.85	18.35–39.07
	HA 4	124	856	14.49	12.20–17.02

* confidence interval.

**Table 3:** Prevalence of *bla*_CTX-M_ of *E. coli* isolated from wild boar by sex, age class, hunting season and hunting area.

Factors		Positive	Total	Prevalence %	95% C.I. *
Sex	Female	96	802	11.97	9.8–14.42
	Male	89	702	12.68	10.31–15.37
Age class	Young	57	345	16.52	12.76–20.87
	Sub-adult	37	368	10.05	7.18–13.59
	Adult	91	791	11.5	9.36–13.94
Hunting season	2017–2018	84	525	16.00	12.97–19.42
	2018–2019	48	381	12.60	9.44–16.35
	2019–2020	53	598	8.86	6.71–11.43
Hunting area	HA 1	66	506	13.04	10.23–16.29
	HA 2	10	63	15.87	7.88–27.26
	HA 3	14	79	17.72	10.04–27.94
	HA 4	95	856	11.10	9.07–13.40

* confidence interval.

**Table 4:** Prevalence of *bla*_TEM_ in *E. coli* isolated from wild boar by sex, age class, hunting season and hunting area.

Factors		Positive	Total	Prevalence %	95% C.I. *
Sex	Female	58	802	7.23	5.54–9.25
	Male	47	702	6.70	4.96–8.80
Age class	Young	27	345	7.83	5.22–11.18
	Sub-adult	19	368	5.16	3.14–7.95
	Adult	59	791	7.46	5.73–9.52
Hunting season	2017–2018	49	525	9.33	6.98–12.15
	2018–2019	21	381	5.51	3.44–8.30
	2019–2020	35	598	5.85	4.11–8.05
Hunting area	HA 1	46	506	9.09	6.73–11.94
	HA 2	4	63	6.35	1.76–15.47
	HA 3	7	79	8.86	3.64–17.41
	HA 4	48	856	5.61	4.16–7.37

* confidence interval.

## Data Availability

The raw data supporting the conclusions of this article will be made available by the authors, without undue reservation.

## Supplementary Materials

The following are available online at https://www.mdpi.com/article/10.3390/ani11071855/s1, Table S1: PCR gene targets, thermal profiles and primer sequences used for the detection of ESBL and AmpC *E. coli*, Figure S1: PCR profiles of ESBL and AmpC *E. coli* genes. Lane 1: negative control; Lane 2: *bla*_CMY_ (1117 bp); Lane 3: *bla*_SHV_ (795 bp); Lane 4: *bla*_TEM_ (1064 bp); Lane 5–9: *bla*_CTX-M_ (group 1, 415 bp; group 2, 552 bp; group 8, 666 bp; group 9, 205 bp; group 25, 327 bp); Lane 10: 100 bp DNA Ladder Invitrogen™, Table S2: Prevalence of *bla*_CMY_ of *E. coli* isolated from wild boar by sex, age class, hunting season and hunting area, Table S3: Prevalence of *bla*_SHV_ of *E. coli* isolated from wild boar by sex, age class, hunting season and hunting area.

## References

[1] Wasyl D., Zając M., Lalak A., Skarżyńska M., Samcik I., Kwit R., Jabłoński A., Bocian Ł., Woźniakowski G., Hoszowski A. (2018). Antimicrobial Resistance in *Escherichia coli* Isolated from Wild Animals in Poland. Microb. Drug Resist..

[2] Graham D.W., Bergeron G., Bourassa M.W., Dickson J., Gomes F., Howe A., Kahn L.H., Morley P.S., Scott H.M., Simjee S. (2019). Complexities in understanding antimicrobial resistance across domesticated animal, human, and environmental systems. Ann. N. Y. Acad. Sci..

[3] Taneja N., Sharma M. (2019). Antimicrobial resistance in the environment: The Indian scenario. Indian J. Med. Res..

[4] Dahms C., Hubner N.O., Kossow A., Mellmann A., Dittmann K., Kramer A. (2015). Occurrence of ESBL-Producing *Escherichia coli* in Livestock and Farm Workers in Mecklenburg-Western Pomerania, Germany. PLoS ONE.

[5] von Salviati C., Laube H., Guerra B., Roesler U., Friese A. (2015). Emission of ESBL/AmpC-producing *Escherichia coli* from pig fattening farms to surrounding areas. Vet. Microbiol..

[6] Agersø Y., Aarestrup F.M. (2013). Voluntary ban on cephalosporin use in Danish pig production has effectively reduced extended-spectrum cephalosporinase-producing *Escherichia coli* in slaughter pigs. J. Antimicrob. Chemother..

[7] Holtmann A.R., Meemken D., Müller A., Seinige D., Büttner K., Failing K., Kehrenberg C. (2021). Wild Boars Carry Extended-Spectrum β-Lactamase- and AmpC-Producing *Escherichia coli*. Microorganisms.

[8] Ewers C., Bethe A., Semmler T., Guenther S., Wieler L.H. (2012). Extended-spectrum β-lactamase-producing and AmpC-producing *Escherichia coli* from livestock and companion animals, and their putative impact on public health: A global perspective. Clin. Microbiol. Infect..

[9] Vale A.P., Cousins C., Tzora A., McCarron M.-T., Green A., Molloy S., Bainbridge J., Leonard F. (2020). Molecular characterization of fecal *Escherichia coli* isolated from zoo animals. J. Zoo Wildl. Med..

[10] Turchi B., Dec M., Bertelloni F., Winiarczyk S., Gnat S., Bresciani F., Viviani F., Cerri D., Fratini F. (2019). Antibiotic susceptibility and virulence factors in *Escherichia coli* from sympatric wildlife of the Apuan Alps Regional Park (Tuscany, Italy). Microb. Drug Resist..

[11] Heuer H., Schmitt H., Smalla K. (2011). Antibiotic resistance gene spread due to manure application on agricultural fields. Curr. Opin. Microbiol..

[12] Tinoco Torres R., Fernandes J., Carvalho J., Cunha M.V., Caetano T., Mendo S., Serrano E., Fonseca C. (2020). Wild boar as a reservoir of antimicrobial resistance. Sci. Total Environ..

[13] Ramanzin M., Amici A., Casoli C., Esposito L., Lupi P., Marsico G., Mattiello S., Olivieri O., Ponzetta M.P., Russo C. (2010). Meat from wild ungulates: Ensuring quality and hygiene of an increasing resource. Ital. J. Anim. Sci..

[14] Miller R.S., Farnsworth M.L., Malmberg J.L. (2013). Diseases at the livestock–wildlife interface: Status, challenges, and opportunities in the United States. Prev. Vet. Med..

[15] Wolny-Koładka K., Lenart-Boroń A. (2018). Antimicrobial resistance and the presence of extended-spectrum beta-lactamase genes in *Escherichia coli* isolated from the environment of horse riding centers. Environ. Sci. Pollut. Res..

[16] Dhama K., Chakraborty S., Kapoor S., Tiwari R., Kumar A., Deb R., Rajagunalan S., Singh R., Vora K., Natesan S. (2013). One World, One Health-Veterinary Perspectives. Adv. Anim. Vet. Sci..

[17] Mateus-Vargas R.H., Atanassova V., Reich F., Klein G. (2017). Antimicrobial susceptibility and genetic characterization of *Escherichia coli* recovered from frozen game meat. Food Microbiol..

[18] Chiari M., Ferrari N., Bertoletti M., Avisani D., Cerioli M., Zanoni M., Alborali L.G., Lanfranchi P., Lelli D., Moreno Martin A. (2015). Long-Term Surveillance of Aujeszky’s Disease in the Alpine Wild Boar (*Sus scrofa*). EcoHealth.

[19] Van Damme I., Garcia-Graells C., Biasino W., Gowda T., Botteldoorn N., De Zutter L. (2017). High abundance and diversity of extended-spectrum beta-lactamase (ESBL) producing *Escherichia coli* in faeces and tonsils of pigs at slaughter. Vet. Microbiol..

[20] Clermont O., Christenson J.K., Denamur E., Gordon D.M. (2013). The Clermont *Escherichia coli* phylo-typing method revisited: Improvement of specificity and detection of new phylo-groups. Environ. Microbiol. Rep..

[21] Woodford N., Fagan E.J., Ellington M.J. (2006). Multiplex PCR for rapid detection of genes encoding CTX-M extended-spectrum (beta)-lactamases. J. Antimicrob. Chemother..

[22] Arlet G., Rouveau M., Philippon A. (1997). Substitution of alanine for aspartate at position 179 in the SHV-6 extended-spectrum β-lactamase. FEMS Microbiol. Lett..

[23] Mabilat C., Goussard S., Sougakoff W., Spencer R.C., Courvalin P. (1990). Direct sequencing of the amplified structural gene and promoter for the extendedbroad-spectrum β-lactamase TEM-9 (RHH-1) of *Klebsiella pneumonia*. Plasmid.

[24] Dierikx C., van Essen-Zandbergen A., Veldman K., Smith H., Mevius D. (2010). Increased detection of extended-spectrum β-lactamase producing *Salmonella enterica* and *Escherichia coli* isolates from poultry. Vet. Microbiol..

[25] Baldo V., Salogni C., Giovannini S., D’Incau M., Boniotti M.B., Birbes L., Pitozzi A., Formenti N., Grassi A., Pasquali P. (2020). Pathogenicity of Shiga Toxin Type 2e *Escherichia coli* in Pig Colibacillosis. Front. Vet. Sci..

[26] Agresti A. (2007). An Introduction to Categorical Data Analysis.

[27] R Core Team (2020). R: A Language and Environment for Statistical Computing.

[28] Poeta P., Radhouani H., Pinto L., Martinho A., Rego V., Rodrigues R., Gonçalves A., Rodrigues J., Estepa V., Torres C. (2009). Wild boars as reservoirs of extended-spectrum beta-lactamase (ESBL) producing *Escherichia coli* of different phylogenetic groups. J. Basic Microbiol..

[29] Alonso C.A., González-Barrio D., Ruiz-Fons F., Ruiz-Ripa L., Torres C. (2017). High frequency of B2 phylogroup among non-clonally related fecal *Escherichia coli* isolates from wild boars, including the lineage ST131. FEMS Microbiol. Ecol..

[30] Plaza-Rodríguez C., Alt K., Grobbel M., Hammerl J.A., Irrgang A., Szabo I., Stingl K., Schuh E., Wiehle L., Pfefferkorn B. (2021). Wildlife as Sentinels of Antimicrobial Resistance in Germany?. Front. Vet. Sci..

[31] Literak I., Dolejska M., Radimersky T., Klimes J., Friedman M., Aarestrup F.M., Hasman H., Cizek A. (2010). Antimicrobial-resistant faecal *Escherichia coli* in wild mammals in central Europe: Multiresistant *Escherichia coli* producing extended-spectrum beta-lactamases in wild boars. J. Appl. Microbiol..

[32] Bonardi S., Cabassi C.S., Longhi S., Pia F., Corradi M., Gilioli S., Scaltriti E. (2018). Detection of Extended- Spectrum Beta-Lactamase producing *Escherichia coli* from mesenteric lymph nodes of wild boars (*Sus scrofa*). Ital. J. Food Saf..

[33] Friese A., Schulz J., Laube H., von Salviati C., Hartung J., Roesler U. (2013). Faecal occurrence and emissions of livestock-associated methicillin-resistant *S. aureus* (laMRSA) and ESbl/AmpC-producing *E. coli* from animal farms in Germany. Berl. Munch. Tierarztl. Wochenschr..

[34] European Surveillance of Veterinary Antimicrobial Consumption (ESVAC) Project. Sales of Veterinary Antimicrobial Agents in 31 European Countries in 2018 (EMA/24309/2020). https://www.ema.europa.eu/en/documents/report/sales-veterinary-antimicrobial-agents-31-europeancountries-2018-trends-2010-2018-tenth-esvac-report_en.pdf.

[35] Scali F., Santucci G., Maisano A.M., Giudici F., Guadagno F., Tonni M., Amicabile A., Formenti N., Giacomini E., Lazzaro M. (2020). The Use of Antimicrobials in Italian Heavy Pig Fattening Farms. Antibiotics.

[36] Jiang F., Wu Z., Zheng Y., Frana T.S., Sahin O., Zhang Q., Li G. (2019). Genotypes and Antimicrobial Susceptibility Profiles of Hemolytic *Escherichia coli* from Diarrheic Piglets. Foodborne Pathog. Dis..

[37] Pereira R.V.V., Siler J.D., Bicalho R.C., Warnick L.D. (2014). In Vivo Selection of Resistant *E. coli* after ingestion of Milk with Added Drug Residues. PLoS ONE.

[38] Livermore D.M., Canton R., Gniadkowski M., Nordmann P., Rossolini G.M., Arlet G., Ayala J., Coque T.M., Kern-Zdanowicz I., Luzzaro F. (2007). CTX-M: Changing the face of ESBLs in Europe. J. Antimicrob. Chemother..

[39] Mrowiec P., Klesiewicz K., Małek M., Skiba-Kurek I., Sowa-Sierant I., Skałkowska M., Budak A., Karczewska E. (2019). Antimicrobial susceptibility and prevalence of extended-spectrum beta-lactamases in clinical strains of *Klebsiella pneumoniae* isolated from pediatric and adult patients of two Polish hospitals. New Microbiol..

[40] Bailey J.K., Pinyon J.L., Anantham S., Hall R.M. (2011). Distribution of the *bla*_TEM_ gene and *bla*_TEM_-containing transposons in commensal *Escherichia coli*. J. Antimicrob. Chemother..

[41] González D., Gallagher E., Zúñiga T., Leiva J., Vitas A.I. (2020). Prevalence and characterization of β-lactamase-producing *Enterobacteriaceae* in healthy human carriers. Int. Microbiol..

[42] Cantón R., Novais A., Valverde A., Machado E., Peixe L., Baquero F., Coque T.M. (2008). Prevalence and spread of extended-spectrum beta-lactamase-producing Enterobacteriaceae in Europe. Clin. Microbiol. Infect..

[43] Buonavoglia A., Leone P., Solimando A.G., Fasano R., Malerba E., Prete M., Corrente M., Prati C., Vacca A., Racanelli V. (2021). Antibiotics or No Antibiotics, That Is the Question: An Update on Efficient and Effective Use of Antibiotics in Dental Practice. Antibiotics.

