# Supplementary material for: ESBL/AmpC-Producing Escherichia coli in Wild Boar: Epidemiology and Risk Factors
Source: Animals (Basel). 2021 Jun 22;11(7):1855. doi: 10.3390/ani11071855 (PMC8300396; doi:10.3390/ani11071855)
Supplement: Supplementary file 1 [file animals-11-01855-s001.zip › Tables.pdf]

Table S1: PCR gene targets, thermal profiles and primer sequences used for the detection of ESBL and AmpC *E. coli*.

Target	Thermal profile		Sequence (5′-3′)	Reference
bla$_{CTX-M-1}$	94°C for 5 min		F: AAAAATCACTGCGCCAGTTC<br>R: AGCTTATTCATCGCCACGTT	Woodford et al. 2005
bla$_{CTX-M-2}$	94°C for 25 s<br>52°C for 40 s<br>72°C for 50 s	X 30	F: CGACGCTACCCCTGCTATT<br>R: CCAGCGTCAGATTTTTCAGG	
bla$_{CTX-M-9}$			F: CAAAGAGAGTGCAACGGATG<br>R: ATTGGAAAGCGTTCATCACC	
bla$_{CTX-M-8/25}$	72°C for 6 min		F: TCGCGTTAAGCGGATGATGC (group 8)<br>F: GCACGATGACATTCGGG (group 25)<br>R: AACCCACGATGTGGGTAGC (group 8/25)	
bla$_{SHV}$	94°C for 2 min<br><br>94°C for 30 s<br>60°C for 30 s<br>72°C for 1 min<br><br>72°C for 4 min	X 30	F: TTATCTCCCTGTTAGCACC<br>R: GATTTGCTGATTTCGCTCGG	Arlet et al. 1997
bla$_{TEM}$	95°C for 5 min<br><br>94°C for 30 sec<br>42°C for 2 min<br>72°C for 45 s<br><br>72°C for 5 min	X 35	F: ATAAAATTCTTGAAGAC<br>R: TTACCAATGCTTAATCA	Mabilat et al. 1990
bla$_{CMY}$	94°C for 3 min<br><br>94°C for 1 min<br>58°C for 1 min<br>72°C for 1 min<br><br>72°C for 10 min	X 25	F: TGATGAAAAAATCGTTATGCTGC<br>R: GCTTTTCAAGAATGCGCCAGG	Dierikx et al. 2010

Table S2: Prevalence of *bla*$_{CMY}$ of *E. coli* isolated from wild boar by sex, age class, hunting season and hunting area.

**Factors**		**Positive**	**Total**	**Prevalence %**	**95% C.I.***
Sex	Female	4	802	0.50	0.14–1.27
	Male	9	702	1.28	0.59–2.42
Age class	Young	4	345	1.16	0.32–2.94
	Sub-adult	1	368	0.27	0.01–1.50
	Adult	8	791	1.01	0.44–1.98
Hunting season	2017-2018	4	525	0.76	0.21–1.94
	2018-2019	3	381	0.79	0.16–2.28
	2019-2020	6	598	1.00	0.37–2.17
Hunting district	HD 1	6	506	1.19	0.44–2.56
	HD 2	0	63	0.00	0.00–5.57
	HD 3	3	79	3.80	0.79–10.70
	HD 4	4	856	0.47	0.13–1.19

*confidence interval.

Table S3: Prevalence of *bla*$_{SHV}$ of *E. coli* isolated from wild boar by sex, age class, hunting season and hunting area.

**Factors**		**Positive**	**Total**	**Prevalence %**	**95% C.I.***
Sex	Female	4	802	0.50	0.14–1.27
	Male	3	702	0.43	0.09–1.24
Age class	Young	1	345	0.29	0.01–1.60
	Sub-adult	3	368	0.82	0.17–2.36
	Adult	3	791	0.38	0.08–1.10
Hunting season	2017-2018	6	525	1.14	0.42–2.47
	2018-2019	1	381	0.26	0.01–1.45
	2019-2020	0	598	0.00	0.00–0.06
Hunting area	HA 1	4	506	0.79	0.22–2.01
	HA 2	0	63	0.00	0.00–5.69
	HA 3	0	79	0.00	0.00–4.56
	HA 4	3	856	0.35	0.07–1.02

*confidence interval.
