# Supplementary figures and images for: ESBL/AmpC-Producing Escherichia coli in Wild Boar: Epidemiology and Risk Factors
Source: Animals (Basel). 2021 Jun 22;11(7):1855. doi: 10.3390/ani11071855 (PMC8300396; doi:10.3390/ani11071855)

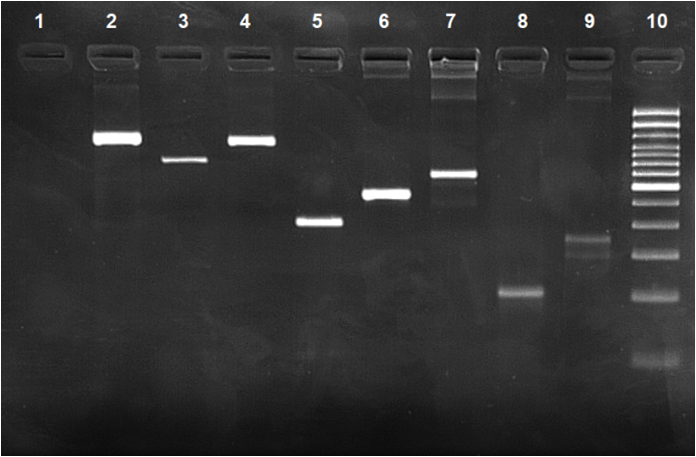

Supplement: Supplementary file 1 [file animals-11-01855-s001.zip › Figure S1.tif]
